# A Case of Mesothelial‐Cell Inclusions in the Mediastinal Lymph Nodes After Lung Cancer Surgery

**DOI:** 10.1002/rcr2.70276

**Published:** 2025-07-16

**Authors:** Hiroyuki Miura, Jun Miura, Shinichi Goto, Tomoko Yamamoto

**Affiliations:** ^1^ Department of Thoracic Surgery Akiru Municipal Medical Centre Tokyo Japan; ^2^ Department of Thoracic Surgery Tomei Atsugi Hospital Kanagawa Japan; ^3^ Department of Respirology Akiru Municipal Medical Centre Tokyo Japan; ^4^ Department of Pathology Tokyo Women's Medical University Tokyo Japan

**Keywords:** lung cancer, mediastinal lymph node, mesothelial‐cell inclusions, video‐assisted thoracoscopic surgery

## Abstract

Mesothelial‐cell inclusion (MCI) is an inclusion of mesothelial cells found in lymph nodes. Although it is a rare condition, differentiating it from lymph node metastasis is essential to avoid a misdiagnosis. We reported, for the first time, a case of MCI after lung cancer surgery. The patient was a 76‐year‐old Japanese man with a history of bilateral radical surgery for lung cancer. Follow‐up computed tomography performed at 7 years after the initial surgery revealed an enlarged right cardiophrenic angle lymph node. It was resected through video‐assisted thoracic surgery, and histopathology revealed non‐dysplastic ductal proliferation. Immunohistochemistry was positive for AE1/3, D2‐40, calretinin, and WT‐1, and negative for CD31, CD34, and factor VIII. Based on these findings, MCI was finally diagnosed. Although the presence of metastasis is the first possible cause for the enlarged lymph nodes in such cases, MCI must also be considered.

## Graphical abstract

We reported, for the first time, a case of mesothelial‐cell inclusion (MCI) after lung cancer surgery.

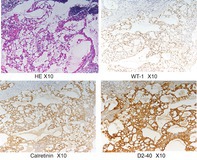

## 1 Introduction

In lung cancer patients, lymph node metastasis must be considered in cases with enlarged mediastinal lymph nodes even if the epithelium is found in those lymph nodes. Mesothelial‐cell inclusions (MCIs), which are a benign condition with lymph node inclusions of the mesothelial cells, were proposed by Brooks et al. in 1990 [1]. They reported two cases of mesothelial cells in the mediastinal lymph nodes mimicking a metastatic carcinoma. Rutty et al. have reported a case of MCIs associated with a malignant lymphoma [2]. Furthermore, they examined 318 mediastinal lymph nodes in 80 patients and found no MCIs. Thus, although this is a rare phenomenon, it is extremely important to differentiate MCIs from lymph node metastasis of the malignant neoplasms for better outcomes. Here, we reported, for the first time, a case of MCIs post‐lung cancer surgery.

## 2 Case Report

A 76‐year‐old Japanese man with a history of bilateral lung adenocarcinoma underwent segmentectomy of the superior segment of the left lung (S6) with hilar lymph node dissection 7 years prior. Additionally, right upper lobectomy with hilar and mediastinal lymph node dissection was conducted 3 years ago. The left tumour, measuring 21 × 12 mm, proved to be a TNM stage of pT1bN0M0 and a pathological stage of IA2 lepidic adenocarcinoma. The right tumour, measuring 16 × 12 × 5 mm in size, was a solid adenocarcinoma with a TNM stage of pT1bN1M0 and a pathological stage of IIB with hilar lymph node involvement. Double primary lung cancer was considered according to the pathological difference. The patient underwent four courses of postoperative adjuvant chemotherapy, comprising cisplatin and vinorelbine. He had a medical history of hypertension and smoking (two packs per day for 50 years), with no remarkable family history.

**FIGURE 1:**
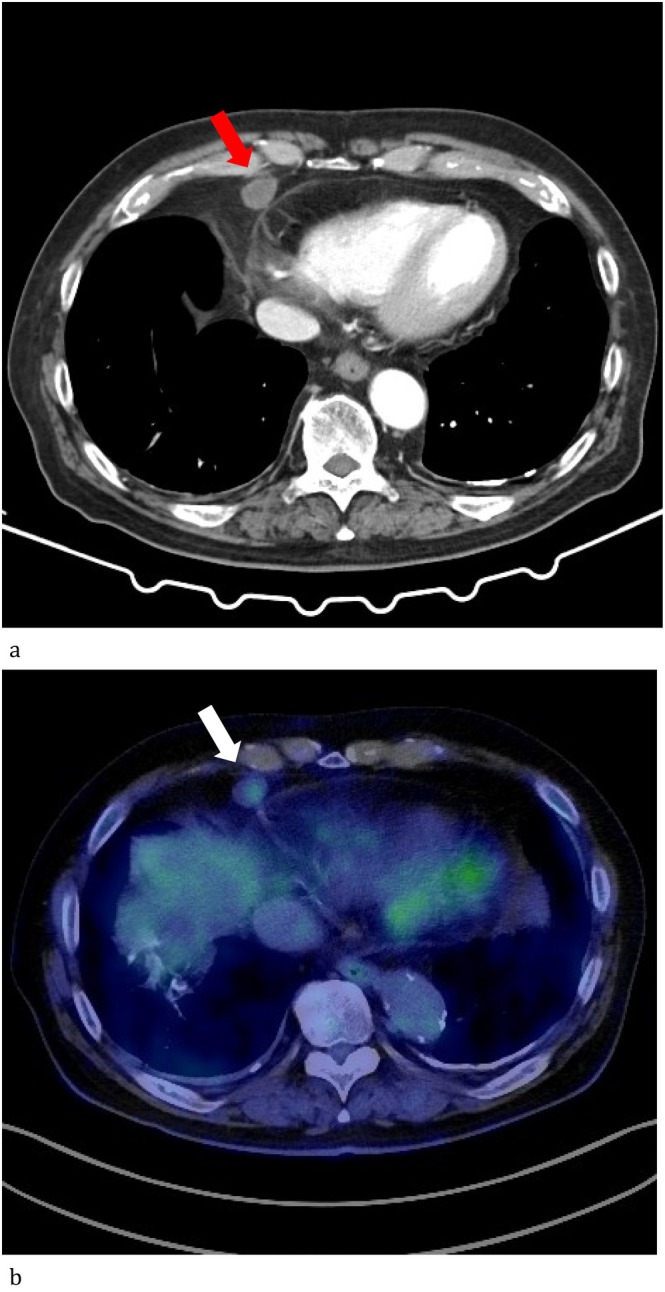
(a) Chest CT. An enlarged right cardiophrenic angle lymph node is seen (orange arrow). (b) Positron emission tomography. A slight fluorodeoxygrucose accumulation was observed in the tumour (white arrow).

**FIGURE 2:**
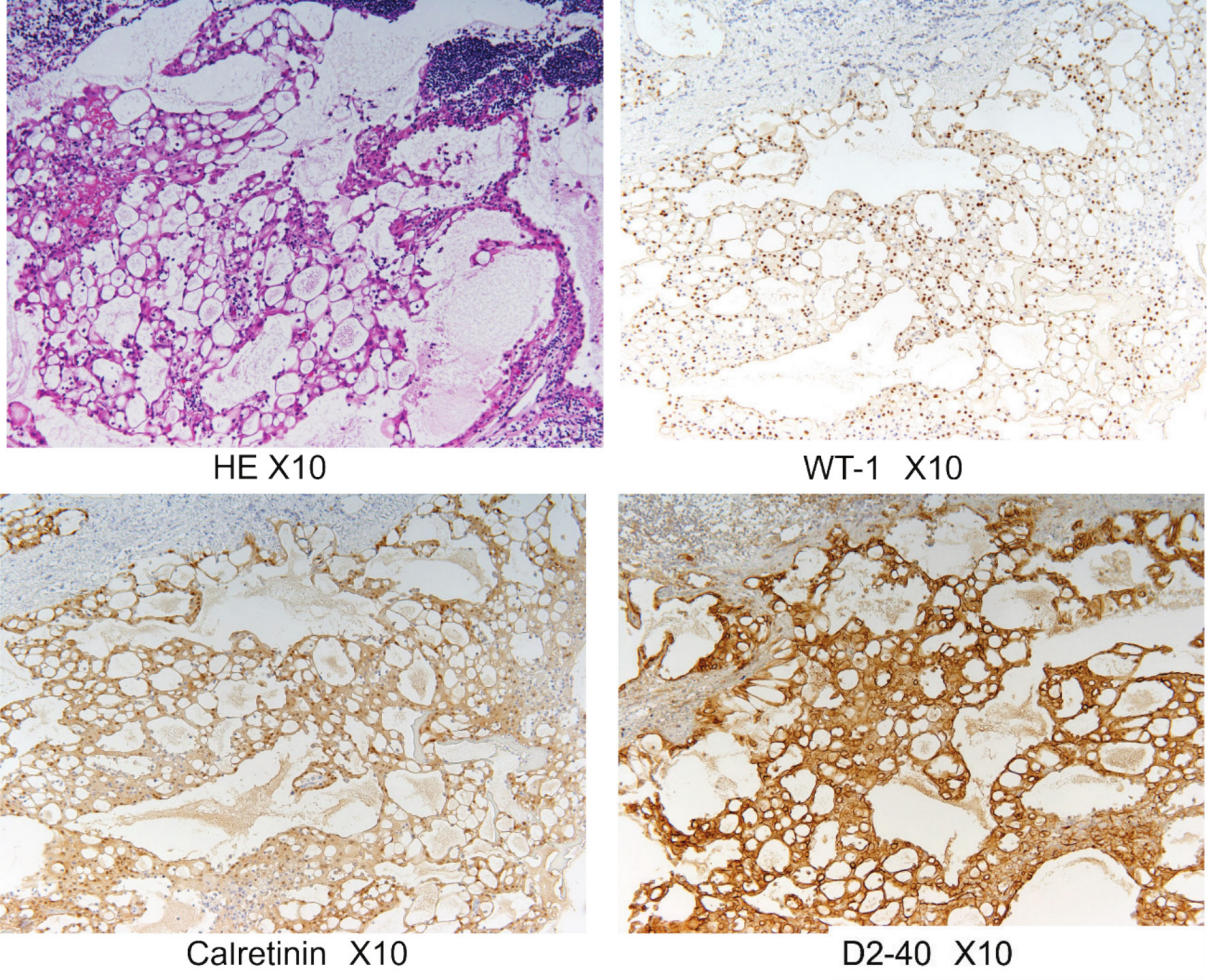
Pathological findings. The tubular proliferation cells were positive for AE1/3, D2‐40, calretinin and WT‐1, and negative for CD31, CD34 and factor VIII.

Seven years after the initial surgery, a swollen right cardiophrenic angle lymph node was detected by follow‐up chest computed tomography (CT) (Figure 1a). Positron emission tomography (PET) revealed fluorodeoxyglucose accumulation in the nodule with a maximum standard uptake value increasing from 2.0 to 2.4 on delay scan. Lymph node metastasis could not be ruled out (Figure 1b). Therefore, this LN was removed under video‐assisted thoracoscopic surgery. The intraoperative diagnosis of the frozen section revealed a ductal proliferation of non‐dysplastic cells in the lymph nodes, making it difficult to know its nature (benign vs. malignant). These tubular proliferation cells were positive for AE1/3, D2‐40, calretinin and WT‐1, and negative for CD31, CD34 and factor VIII (Figure 2). MCI was diagnosed based on the immunohistochemistry results. The patient had a generally good condition with no recurrence over 2 years postoperatively.

## 3 Discussion

The mechanism of occurrence of MCI was unclear, but the following three theories have been reported [1, 2]: (1) it is caused by the metaplastic proliferation of the peritoneal mesothelium derived from the foetal coelomic epithelium; (2) foetal tissue is entrapped into the lymph nodes during embryonal development and (3) mesothelial cells stimulated by inflammation or neoplasms migrate into the submesothelial lymphatic vessels and are taken up into the lymph nodes. The latter is thought to occur when the exudate induces mesothelial cells to migrate into the lymphatic vessels. This is the most plausible hypothesis for the cause of our cases.

The first theory mentioned above occurred in the female pelvic or peri‐aortic lymph nodes and was associated with endosalpingiosis, salpingitis and cervical and ovarian malignancy. The second one was observed as a salivary or breast tissue. Although postoperative pleural effusion is a common phenomenon seen mostly in all patients, it is unknown why MCIs only occur in a limited number of patients. Perhaps the mesothelial‐cell migration is a common phenomenon; however, they could be quickly eliminated in most patients.

It is extremely important to distinguish these MCIs from lymph node metastasis of malignant neoplasms. Clinically, it is necessary to carefully evaluate the neoplastic diseases that can potentially be primary lesions. However, it is potentially difficult in cases where the patients have received cancer treatment as in our current reported case. One of the key points that helped in excluding metastases in our case is that the lymph node‐containing MCIs were not in the drainage pathway of lymphatic metastasis of the resected cancer. Although the absence of atypia is a pathological differentiating point, the immunohistochemical positivity for CK7 or pan cytokeratin makes it difficult to distinguish them from metastasis. However, WT1 and D2‐40 positivity and p63, TTF1, PAX8, MART1 and SOX10 negatively established the mesothelial origin [3]. Furthermore, the tumour glycoprotein antibody Ber EP4 is useful in the differentiation between malignant mesothelioma and adenocarcinoma [4]. However, the differentiation between metastasis from malignant mesothelioma and MCIs is extremely difficult because of the considerable clinical and pathological overlap between these two conditions [5]. This differentiation is easy in the absence of malignant mesothelioma and malignant pleural effusion, and is potentially difficult in the presence of one or both of them. The patient's clinical course is also important for the disease differentiation in these cases, in addition to the pathological findings, including the presence or absence of cellular atypia and mitotic figures.

In such cases, biopsies should be performed actively to confirm the presence or absence of metastasis to ensure accurate decision making. We think that it is important to consider the possibility of MIC occurrence in cases of enlarged lymph nodes with pleural effusion or ascites, especially in patients with any malignant tumours. If the surgery was hesitated due to the patient's condition, and the enlarged lymph nodes are not in the drainage pathway for metastasis of the malignant tissue, the LNs could be followed up with CT and/or PET.

## Author Contributions

Hiroyuki Miura and Shinichi Goto helped in the conception and design of the work and the acquisition, analysis or interpretation of data for the work. Jun Miura drafted the work and revised it critically for important intellectual content. Tomoko Yamamoto pathologically diagnosed the tumour. All authors revised the final version of this manuscript and approved it to be published.

## Consent

Appropriate written informed consent was obtained from the patient for publication of this case report and the accompanying images.

## Conflicts of Interest

The authors declare no conflicts of interest.

## Data Availability

Data sharing not applicable to this article as no datasets were generated or analysed during the current study.
